# Pharmacological Investigation of CC-LAAO, an L-Amino Acid Oxidase from *Cerastes cerastes* Snake Venom

**DOI:** 10.3390/toxins13120904

**Published:** 2021-12-16

**Authors:** Zaineb Abdelkafi-Koubaa, Ines ELBini-Dhouib, Soumaya Souid, Jed Jebali, Raoudha Doghri, Najet Srairi-Abid, Khadija Essafi-Benkhadir, Olivier Micheau, Naziha Marrakchi

**Affiliations:** 1 Laboratoire des Biomolécules, Venins et Applications Théranostiques (LR20IPT01), Institut Pasteur de Tunis, Université de Tunis El Manar, Tunis 1002, Tunisia; ines.bini@pasteur.tn (I.E.-D.); jed.jebali@pasteur.tn (J.J.); najet.abidsrairi@pasteur.tn (N.S.-A.); naziha.marrakchi@pasteur.tn (N.M.); 2 Laboratoire d’Epidémiologie Moléculaire et de Pathologie Expérimentale (LR16IPT04), Institut Pasteur de Tunis, Université de Tunis El Manar, Tunis 1002, Tunisia; souid@ulm.edu (S.S.); khadija.essafi@pasteur.tn (K.E.-B.); 3 School of Basic Pharmaceutical and Toxicological Sciences, College of Pharmacy, University of Louisiana at Monroe, 1800 Bienville Drive, Monroe, LA 71201, USA; 4 Département d’Anatomie Pathologique, Institut Salah Azaiez, Bab Saadoun, Tunis 1006, Tunisia; raoudha.doghri@gmail.com; 5 Lipides Nutrition Cancer, INSERM-UMR 1231, Université de Bourgogne Franche-Comté, UFR Science de Santé, 7 Bd Jeanne d’Arc, 21000 Dijon, France; omicheau@u-bourgogne.fr; 6 Faculté de Médecine de Tunis, Université de Tunis El Manar, Tunis 1068, Tunisia

**Keywords:** SV-LAAOs, envenomation, toxicity, apoptosis, glioblastoma cells

## Abstract

Snake venom proteins, which are responsible for deadly snakebite envenomation, induce severe injuries including neurotoxicity, myotoxicity, cardiotoxicity, hemorrhage, and the disruption of blood homeostasis. Yet, many snake-venom proteins have been developed as potential drugs for treating human diseases due to their pharmacological effects. In this study, we evaluated the use of, an L-amino acid oxidase isolated from *Cerastes cerastes* snake venom CC-LAAO, as a potential anti-glioblastoma drug, by investigating its in vivo and in vitro pharmacological effects. Our results showed that acute exposure to CC-LAAO at 1 and 2.5 µg/mL does not induce significant toxicity on vital organs, as indicated by the murine blood parameters including aspartate transaminase (AST), alanine transaminase (ALT), lactate dehydrogenase (LDH) activities, and creatinine levels. The histopathological examination demonstrated that only at high concentrations did CC-LAAO induce inflammation and necrosis in several organs of the test subjects. Interestingly, when tested on human glioblastoma U87 cells, CC-LAAO induced a dose-dependent apoptotic effect through the H_2_O_2_ generated during the enzymatic reaction. Taken altogether, our data indicated that low concentration of CC-LAAO may be safe and may have potential in the development of anti-glioblastoma agents.

## 1. Introduction

The World Health Organization (WHO) has included envenomation by snake bites in their list of neglected tropical diseases because it has been associated with significant morbidity and mortality, especially in tropical and subtropical areas [1]. Snake venoms are mainly composed of enzymes such as phospholipases A2 (PLA2s), metalloproteases (SVMPs), serine-proteases (SVSPs), hyaluronidases, and L-amino acid oxidases (LAAOs). The combined action of this family of proteins found in the same venom likely results in additive or synergistic antagonizing effects that induce a wide range of toxic effects [2]. Nevertheless, each protein has distinct pharmacological effects with high specificity and affinity for cells and tissues [3].

Systemic hemodynamic disturbances have been mostly attributed to SVSPs [4]. PLA2s have been shown to induce local myonecrosis and lymphatic vessel damage [5], and SVMPs are responsible for local hemorrhage, extracellular matrix degradation, blistering, and skin necrosis [6]. In addition, hyaluronidases and other proteases have been identified as spreading factors that act by degrading hyaluronic acid and other extracellular matrix constituents [7]. Furthermore, SV-LAAOs are found in high concentrations that vary between snake species and may contribute to the toxicity of the venom. LAAO (LAAOs, EC 1.4.3.2) is an FAD-containing dimeric enzyme that stereospecifically deaminates an L-amino acid to an α-keto acid with the concomitant production of hydrogen peroxide (H_2_O_2_) and ammonia [8]. The specific role of SV-LAAOs in venom toxicity and its consequences to the prey are not yet clear [8]. Nevertheless, a high correlation between in vitro LAAO activity and in vivo necrosis was reported in the *bothropic* venom, suggesting the involvement of LAAO in the dermonecrosis caused by the crude venom [9]. Moreover, Costal-Oliveira et al. demonstrated that LAAO from *Bothrops atrox* snake venom, triggers autophagy, apoptosis, and necrosis in normal human keratinocytes [10]. This finding contributes to a better understanding of LAAOs’ mechanisms of action and provides insights into its contribution to localized tissue necrosis during envenomation. 

Interestingly, the induction of apoptosis in tumor cells has been one of the most important mechanisms of anti-cancer agents. Therefore, SV-LAAOs have been studied for their anti-cancer potential through cytotoxic and apoptotic activities in several cancer cell lines [11]. However, the cytotoxic mechanism remains poorly understood. In this regard, many hypotheses have stipulated that the accumulated H_2_O_2_ generated during the LAAO catalytic activity leads to oxidative stress and cell death [5]. In fact, numerous studies have demonstrated a decrease in the cytotoxic effect of SV-LAAO upon exposure to catalase enzyme or glutathione (GSH) peptide, which inhibited the H_2_O_2_ activity [8]. Another study supporting this hypothesis noticed that H_2_O_2_ is a mediator of apoptosis by acting directly on the oxidative cell metabolism [12]. Hence, the potential of SV-LAAOs as an apoptotic agent may lie in a deeper understanding of its mechanism of action.

In this context, we previously identified a LAAO from the horned Tunisian desert viper, *Cerastes cerastes*, designated CC-LAAO [13]. This 115 kDa homodimeric glycoprotein showed antibacterial effects as well as high cytotoxicity against several cancer cell lines including human breast cancer cells (i.e., MDA-MB-231 and MCF-7), rat adrenal medulla pheochromocytoma cells (PC12), and a murine melanoma cell line (B16F10) with negligible effects on erythrocytes and human peripheral blood mononuclear cells (PBMC) [14]. 

The present study aimed to improve the understanding of the toxicological mechanisms and pharmacological potential of CC-LAAO on an in vivo model as well as its anti-tumor effect on human glioblastoma U87 cells.

## 2. Results

### 2.1. In Vivo Study

#### 2.1.1. General Observation

To determine the safety concentration of CC-LAAO, which could eventually be used for therapeutic purposes, six male Swiss albino mice, per group, were injected by intraperitoneal route with different concentrations of CC-LAAO, and the physiological observations were reported to evaluate their general health status.

As an initial step of general observation, we found that 500 µg/mL of CC-LAAO induced 100% mortality 2 h post-injection, whereas no signs of toxicity were recorded in the groups of mice injected with 1 µg/mL (M1); 2.5 µg/mL (M2); 10 µg/mL (M3); or 50 µg/mL (M4) after 24 h post-injection. In addition, we observed normal ingestive behavior with no evidence of skin reaction or sensory nervous system response, as compared to the negative control animals (CTR−). The same results were observed with the injection of 6 µg (half of LD_50_) of the *Cerastes cerastes* venom (CTR+).

#### 2.1.2. Effect of Acute Injection of CC-LAAO on Blood Biochemical Parameters

We analyzed blood parameters such as aspartate transaminase (AST), alanine transaminase (ALT), lactate dehydrogenase (LDH) activities, and creatinine levels, which are considered to be markers of main organ toxicity. In fact, AST and ALT are markers for liver toxicities while a high level of LDH implies general toxicity related to the liver, heart, kidneys, and skeleton muscles. In addition, elevated creatinine levels are indicators of renal damage and muscle injury.

**Figure 1:**
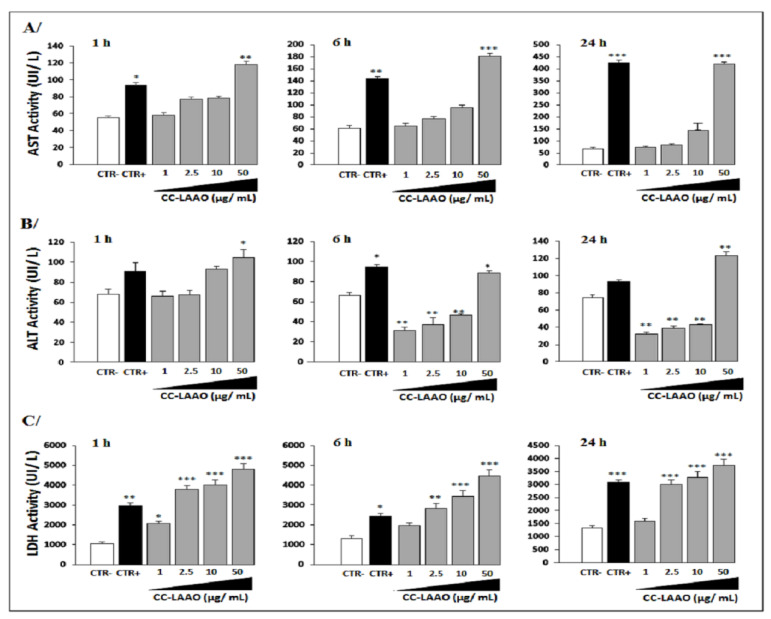
Hepatic enzymes activity (AST, ALT, and LDH) in plasma of mice. (**A**) Plasma aspartate aminotransferase (AST) activity. (**B**) Plasma alanine aminotransferase (ALT) activity. (**C**) Plasma lactate dehydrogenase (LDH) activity. Enzymatic activity was expressed in international units per liter (UI/L). Blood samples were collected from facial veins of each animal after 1 h, 6 h, and 24 h. Plasma were obtained by centrifugation at 3000× *g* for 10 min and stored at −20 °C until use. Enzymatic activity was expressed in international units per liter (UI/L). CTR−: negative control group; CTR+: mice treated by crude venom (the half of LD_50_); M1: mice received CC-LAAO molecules at 1 µg/mL; M2: mice received CC-LAAO molecules at 2.5 µg/mL; M3: mice received CC-LAAO molecules at 10 µg/mL; and M4: mice received CC-LAAO molecules at 50 µg/mL. Experiments in triplicate were performed for each group of mice. *, **, and *** denote *p* < 0.05, 0.01, and 0.001, respectively.

Our results showed that AST blood levels were significantly increased in the animals injected with the total venom or the high concentrations of CC-LAAO molecules (50 µg/mL), and AST levels remained high up to 24 h after administration, as compared to those with low concentrations, in which their AST blood levels decreased rapidly by 6 h post-injection (Figure 1A).

Statistically significant differences of blood ALT activity were found between the animal groups treated with CC-LAAO at 1 µg/mL (M1), 2.5 µg/mL (M2), and 10 µg/mL (M3) as well as the CTR− group (Figure 1B). In contrast, this activity increased in the CTR+ (animals treated with 6 µg (the half of LD_50_) of *Cerastes cerastes* venom) and the M4 (50 µg/mL) mice groups (*p* < 0.01). 

Additionally, mean LDH values were increased in all treated groups (Figure 1C). Particularly, the animals treated with CC-LAAO at 50 µg/mL had LDH levels well above the IDEXX reference range for blood LDH.

In contrast, creatinine levels, as a kidney acute injury marker, were evaluated in the control and treated mice. Our statistical analysis demonstrated a significant increase in CC-LAAO-treated mice, as compared to mock-treated animals (Table 1). 

#### 2.1.3. Effect of Acute Injection of CC-LAAO in Mouse Tissues

**Figure 2:**
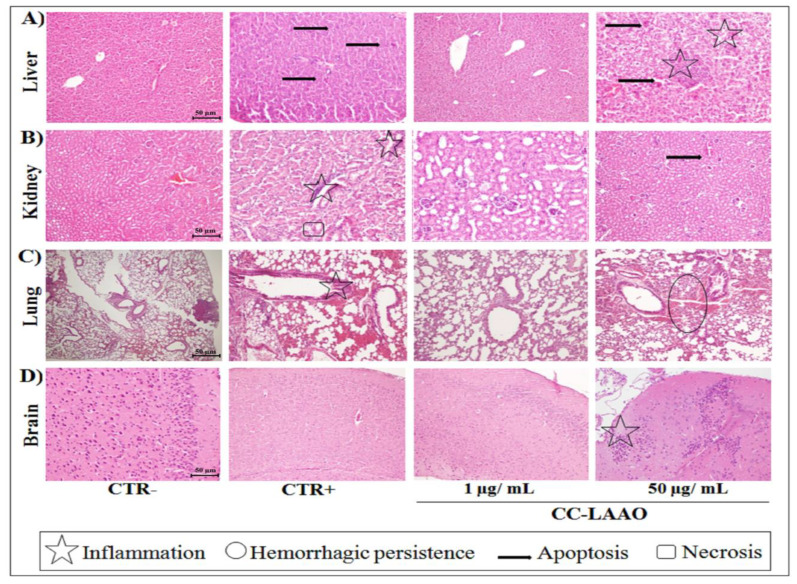
Histopathological examination of various organs of the mouse in toxicity study. (**A**) Liver. (**B**) Kidney. (**C**) Lung. (**D**) Brain. Mice were euthanized, and their organs were excised and fixed in 10% buffered formalin. Organs were stained with hematoxylin and eosin (HE) dye. (CTR−): negative control group; (CTR+): mice treated by crude venom (half of LD_50_); (M1): mice received CC-LAAO molecules at 1 µg/mL; and (M4): mice received CC-LAAO molecules at 50 µg/mL.

The histopathological examination of murine livers stained with hematoxylin and eosin (HE) dye showed that total venom and CC-LAAO injected at 10 and 50 µg/mL led to various degrees of damage in the liver, as compared to the CTR− group, which appeared normal. The effect of CC-LAAO was dose-dependent since we noticed a slight inflammatory cell infiltration in the livers of the M3 (10 µg/mL) group (data not shown), whereas the severe lesions in the M4 group (50 µg/mL) highlighted necrosis and inflammatory cell infiltration. In addition, hepatic injury was less severe in the M1 (1 µg/mL) group (Figure 2A).

Furthermore, the histological analysis of the HE-stained tissues revealed that the mice treated with total venom exhibited severe renal pathological lesions (Figure 2B), which was indicated by widespread tubular necrosis and degeneration as well as cellular swelling and inflammatory cell infiltration in renal tissues. These damages were only observed in the highest concentration of CC-LAAO (50 µg/mL). 

The representative photomicrographs of stained lungs (Figure 2C) showed that the tissues from treated mice as well as those of the CTR− and CTR+ groups had a typical structural appearance. Indeed, neither erosion rupture nor tissue abnormalities were noticed. However, we noticed hemorrhagic persistence in the M3 (data not shown) and M4 groups (Figure 2C).

We also noticed inflammation in the brains of the M4 mice while no evidence of tissue damage was observed in the other mice groups (Figure 2D).

#### 2.1.4. Edema-Inducing Activity

**Figure 3:**
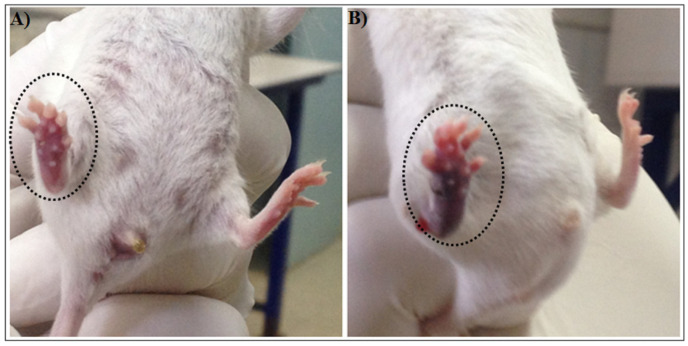
Edema-inducing activity of CC-LAAO. The CC-LAAO induced edema in paw pads (right) of mice at the doses of 4 µg (**A**) and 16 µg (**B**).

CC-LAAO induced edema in the paw pads of the mice and produced a rapid, considerable increase in the paw volume with a minimum edema dose (MED) of 8 μg/paw. CC-LAAO at 4 µg produced an edema that peaked 1 h after the injection, and the edema was intensely sustained for up to 6 h (Figure 3A). At a concentration of 16 μg, the edema was sustained for more than 24 h (Figure 3B).

The same volume of vehicle (saline 0.9% *v*/*v*) was injected into the contralateral paw of a mouse as a negative control. After 2 h, the thickness of the swelling paws was measured with a vernier caliper.

#### 2.1.5. Hemorrhagic Activity

**Figure 4:**
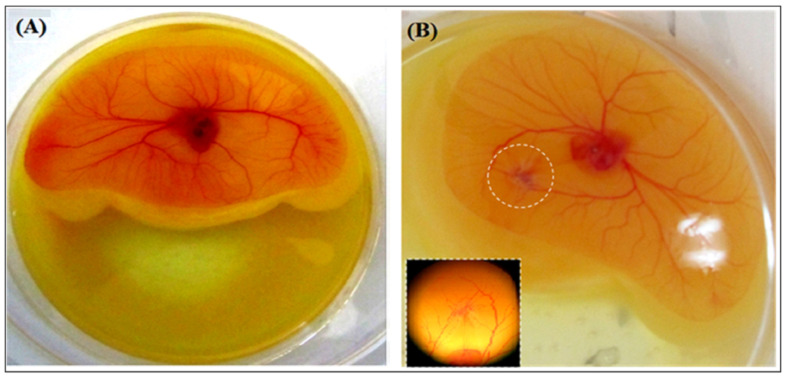
Hemorrhagic activity of CC-LAAO. (**A**) Embryo treated with saline (0.9%). (**B**) Embryo treated with CC-LAAO (50 µg/mL). Insert: 10× magnification around the disc. Discs of 2 mm diameter (Whatman) were impregnated with various concentrations of CC-LAAO. The discs were placed on the yolk-sac membrane over a major bilateral vein. Negative control was performed with the buffered saline solution. After 6 h, hemorrhagic corona formation was observed and photographed with a digital camera at 10× magnification.

To determine the hemorrhagic activity of CC-LAAO, different concentrations were studied on the vasculature development in a chicken chorioallantoic membrane (CAM) model. Results showed that CC-LAAO, only at a high concentration (50 µg/mL), induced a slight hemorrhage (Figure 4). 

### 2.2. CC-LAAO Effects on U87 Cell Viability

**Figure 5:**
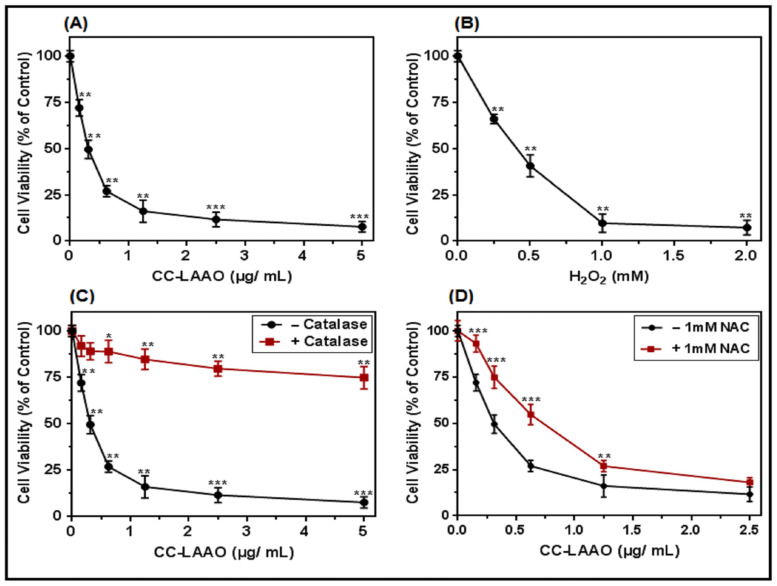
CC-LAAO inhibits U87 cell viability. (**A**) MTT assay indicated that CC-LAAO treatment for 24 h inhibited U87 cell viability depending on the dosage. (**B**) Exogenous H_2_O_2_ inhibited U87 cell viability. (**C**) Influence of catalase on CC-LAAO cytotoxicity on U87. (**D**) U87 cells were incubated in the presence or absence of NAC for 2 h and treated with CC-LAAO for 24 h. Cell viability was assessed via MTT assay. The absorbance was measured at 560 nm. Results were normalized to each control by percentage and represented as mean ± SE of the three independent experiments. Triplicate experiments were performed for each group. *, **, and *** denote *p* < 0.05, 0.01, and 0.001, respectively.

To further analyze the effect of CC-LAAO, we assessed its inhibitory activity in vitro on U87 cells. Our results showed that CC-LAAO diminished the viability of the U87 cells in a concentration-dependent manner, yielding an IC_50_ value of 0.3 µg/mL (2.6 nM) (Figure 5A). The exogenous H_2_O_2_ administration yielded an IC_50_ value of ≃0.4 mM (Figure 5B). To verify if the generated H_2_O_2_ during the CC-LAAO catalytic activity would lead to cell viability inhibition, the SV enzyme was combined with a catalase (1 mg/mL), an efficient scavenger of H_2_O_2_. As demonstrated in Figure 5C, the catalase drastically diminished the cytotoxic effect of CC-LAAO on the U87 cells. Similarly, albeit to a much lesser extent, the ROS-scavenger N-acetylcysteine (NAC) partially reduced the CC-LAAO-induced cytotoxicity on the U87 cells (Figure 5D). These results suggest that CC-LAAO inhibited U87 cell viability through H_2_O_2_ production.

### 2.3. Apoptosis-Induction Effect

**Figure 6:**
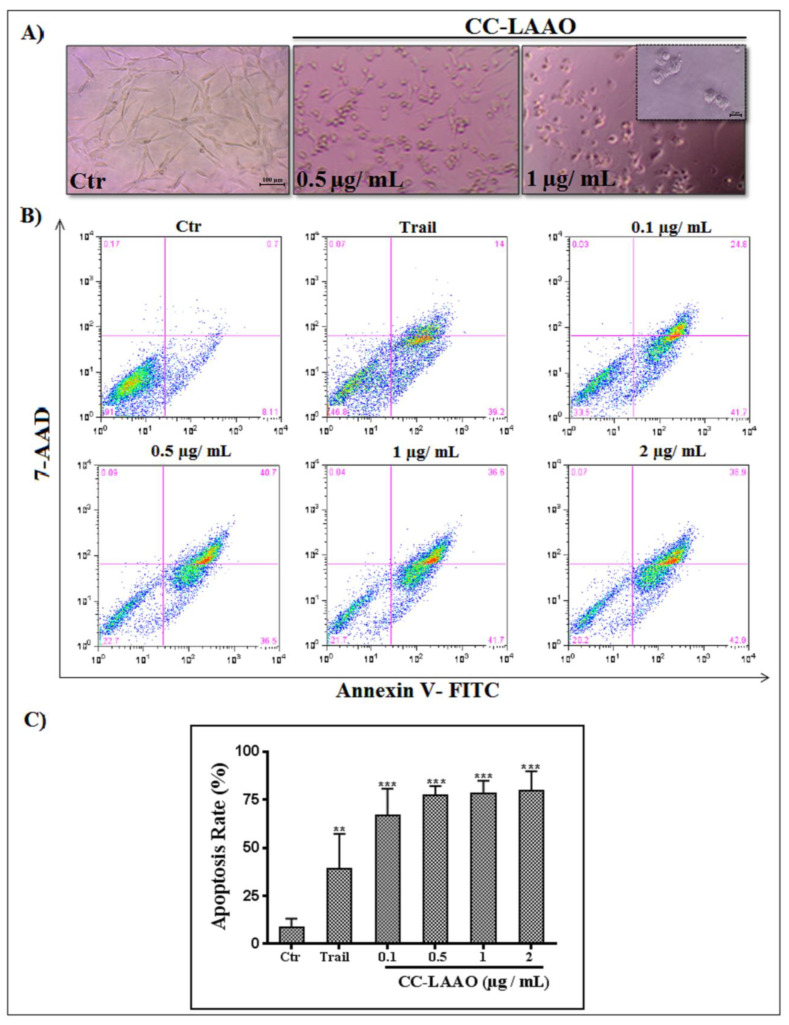
Apoptotic effect of CC-LAAO. (**A**) Influence of CC-LAAO treatment on U87 cell morphology. U87 cell morphology observation in the absence (Ctr) and the presence of CC-LAAO: 0.5 µg/mL; and 1 µg/mL. Cell images were taken using an inverted-light microscope at magnifications of 10× and 40× (Insert). The results are representative of the three independent experiments. (**B**) Flow cytometric analysis of CC-LAAO effect on U87 cells. Mock-stimulated culture cells were used as negative controls, and the positive controls received TNF-related apoptosis-inducing ligand (TRAIL) (0.1 µM for 6 h). Cells treated with CC-LAAO (0.1–2 µg/mL) for 24 h were double stained with annexin V/7-AAD, as reported in the Methods and Materials section, and analyzed by flow cytometry. The C1 area represents cell necrosis, the C2 area represents late apoptosis, the C3 area represents the living cells, and the C4 area represents early apoptotic cells. (**C**) Apoptosis rate from flow cytometry. The apoptosis rate is the number of apoptotic cells divided by the number of total cells × 100. The results are representative of three independent experiments. *, **, and *** denote *p* < 0.05, 0.01, and 0.001, respectively, versus negative control.

The morphological alterations of the U87 cells treated with CC-LAAO were analyzed under a phase-contrast microscope (Figure 6A). The mock-treated cells were uniform in size, appeared elongated, and attached smoothly to the plate surface. After CC-LAAO treatment, the cells showed severe changes in their general morphology. Reduced cell volume and density were clearly observed. The cell shape changed from elongated to round with increased intercellular spaces. In addition, the cells detached from the culture plates and floated in the medium.

To better understand how CC-LAAO induces cell death, we labeled the U87 cells with annexin V-FITC and 7-AAD. The population of the early apoptotic cells was marked with annexin V-FITC (AV^+^/7-AAD^−^). The necrotic cells were AV^−^/7-AAD^+^, and late-apoptotic cells were labeled with both markers (AV^+^/7-AAD^+^). In agreement with the results of the cell viability assay (Figure 6), CC-LAAO at concentrations as low as 0.1 µg/mL induced apoptosis in the U87 cells at levels well above TRAIL, a cytokine known to trigger cell death by binding to its membrane-bound agonist receptors either through the activation of caspases or the necroptosome (Figure 6B,C).

**Figure 7:**
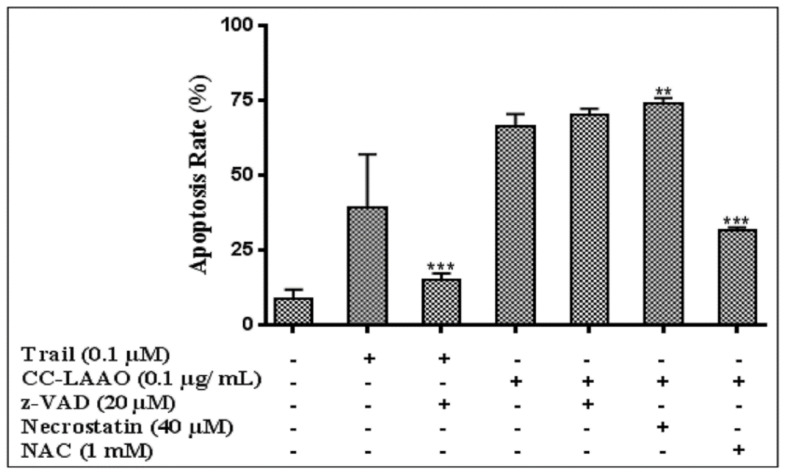
Influence of z-VAD-fmk, necrostatin, or N-acetylcysteine (NAC) on the apoptotic effect induced by CC-LAAO on U87 cells. Cells were pre-treated with 20 µM z-VAD-fmk or 40 µM necrostatin or 1 mM N-acetylcysteine (NAC) for 2 h and treated with CC-LAAO (0.1 µg/mL) for 24 h, double-stained with annexin V/7-AAD and analyzed by flow cytometry. *, **, and *** denote *p* < 0.05, 0.01, and 0.001, respectively, versus 0.1 µg/mL.

We then investigated the role of caspase activation, necrotic cell death, and oxidative stress in CC-LAAO-induced apoptosis in the U87 cells by pre-treating the cells with the pan-caspase inhibitor, z-VAD-fmk (20 µM), the necrostatin (40 µM), or NAC (1 mM) before adding the enzyme (0.1 µg/mL). Interestingly, whereas neither Z-VAD-fmk nor necrostatin prevented the apoptotic effect of CC-LAAO on the U87 cells, inhibiting ROS production with NAC impaired CC-LAAO-induced apoptosis (Figure 7).

This result suggested that the induced effects in the U87 cells were independent of caspase activation and were associated with oxidative stress.

## 3. Discussion

The *Cerastes cerastes* viper is a common snake found in Middle Eastern and African deserts and belongs to the Viperidae family [15]. In Tunisia, the sand viper *Cerastes cerastes* is widespread in desert regions and highlands and represents one of the most dangerous vipers. Its bite induces a complex symptomatology that often leads to functional or vital complications, and possibly even death, of the bitten victim [16]. Several active biomolecules have been isolated from this venom, including PLA2 [17], serine proteinases [18] and disintegrins [19]. 

As one of the major protein (enzyme) components of this venom, CC-LAAO may play an important role in its toxicity and biological activities. Indeed, it has been reported that SV-LAAOs are involved in edema, hemolysis, and myotoxicity, which may also contribute to the development of envenomation symptoms [8,20]. However, extensive studies have revealed that SV-LAAOs have considerable cytotoxic effects, particularly on cancer cells, via the induction of apoptosis, cell cycle arrest, and, consequently, the suppression of cell proliferation [5,21]. Although many authors have investigated the mode of action of SV-LAAOs, most of the hypotheses were based on the accumulated H_2_O_2_ generated during the LAAO catalytic activity, which leads to oxidative stress [5,22]. However, the detailed mechanisms are still unclear. In the present study, we pioneered an investigation of the toxicological mechanisms of CC-LAAO in an experimental in vivo model, and we evaluated the cytotoxic and proapoptotic effects of this protein on U87 human glioblastoma cells.

For an envenoming-model study, five distinct routes can be used for venom injection: (i) intracerebroventricular (ICV), (ii) intramuscular (IM), (iii) intravenous (IV), (iv) intraperitoneal (IP), and (v) subcutaneous (SC). In this study, we used the IP route to investigate CC-LAAO lethality and its acute effects [23]. Our results showed that at low concentrations, CC-LAAO did not exhibit lethal toxicity when injected via IP route. This result was consistent with previous studies that reported several SV-LAAOs exhibited moderate toxicity in mice with LD_50_ of approximately 5–9 μg/g. The LD_50_ of LAAO has usually been higher than that of the corresponding venom, and therefore, the LAAO has not been considered a major lethal component of the venom as the enzyme usually constitutes about 5% of the venom dry weight [24].

In the second phase, the effects of CC-LAAO on the function of vital organs, such as the liver and the kidneys, was assessed as they are the primary metabolic targets of any toxic drug. In our data, we found that acute exposure to high concentrations of CC-LAAO (10 and 50 µg/mL) affected significant blood parameters of the mice, including ALT, AST, and LDH values as well as creatinine levels, as compared to non-treated mice. Indeed, CC-LAAO at high concentration was able to induce liver toxicity and renal changes in the experimental model. However, low concentrations of CC-LAAO were found to be non- toxic and safe in mice, suggesting that this molecule may be of potential pharmacological interest. This finding was in accordance with previous studies revealing that SV-LAAO exhibited low systemic toxicity [25,26,27].

Moreover, the histopathological examination demonstrated that, at high concentrations (50 µg/mL), CC-LAAO induced inflammation and necrosis in several organs of the mice.

Our results corroborated those of Wei et al., which showed that ABU-LAO at 10 µg/mL induced liver-cell necrosis and stimulated lymphocytes and monocytes to release IL-6, IL-2, IL-12, and T cells when injected intravenously to BALB/c mice [28].

Furthermore, similar to other SV-LAAOs, CC-LAAO induced edema in the mice [29,30,31]. For instance, BF-LAAO, isolated from *Bungarus fasciatus* venom, induced rat-paw edema and myotoxicity on the gastrocnemius muscles of mice by causing severe myofibrosis, myoedema, inflammatory cell accumulation, and myolysis [26]. The induction of edema may be due to (i) the generation of H_2_O_2_, (ii) the subsequent inflammatory response mediated by the release of autacoids or eicosanoids, such as prostaglandin, and/or (iii) the metabolism of cyclooxygenases [32].

However, CC-LAAO showed slightly hemorrhagic activity at high concentration (50 µg/mL), as comparable to that of batrox-LAAO from *Bothrops atrox* venom [33]. Until recently, only ACL-LAO from *Agkistrodon contortrix laticinctus* venom, with a minimum dose of 10 µg, was thought to be able to induce hemorrhaging in mice. Therefore, the hemorrhagic activity of SV-LAAO has been considered low, as compared to that of snake venom metalloproteinases, which usually range from 0.02 to 10 µg [34].

Based on the present study’s findings that low concentrations of CC-LAAO were safe in mice, we investigated its potential effect on U87 human glioblastoma cells. Interestingly, CC-LAAO reduced cell viability in a concentration-dependent manner, with an IC_50_ of 0.3 µg/mL (2.6 nM). Previously, we reported that CC-LAAO did not affect the viability of PBMCs from healthy donors and induced less than 10% hemolysis on erythrocytes even at higher concentrations [14].

The IC_50_ of CC-LAAO was found to be seven-times lower than that of OHAP-1, an LAAO isolated from *Protobothrops flavoviridis* venom, which has exhibited cytotoxic effects against malignant glioma cell lines including C6 (rat), RBR 17T, and U251 cells (human) with IC_50_ values of 1.9 µg/mL, 2.48 µg/mL, and 2.1 µg/mL, respectively [35].

Our results showed that the effect of CC-LAAO was mediated, at least in part, by the action of the hydrogen peroxide generated during the enzymatic reaction. Indeed, co-treatment with catalase (as a scavenger of hydrogen peroxide) significantly diminished the cytotoxicity of both the enzyme and the exogenous H_2_O_2_. Our findings were in accordance with those of Izidoro et al., which demonstrated that H_2_O_2_ was the major agent responsible for the cytotoxic effect of SV-LAAOs [8]. In addition, we found that the IC_50_ of CC-LAAO (2.6 nM) was 10^5^-fold lower than that of exogenous H_2_O_2_ (IC_50_ ~ 0.4 mM). According to our actual results as well as a previous study [14], we suggested that the enzyme binds to the cell surface and generates a highly localized concentration of H_2_O_2_ in the binding site that may elicit a potent cytotoxic effect.

Furthermore, cells treated with CC-LAAO showed morphological changes, which were observed by an inverted-phase contrast microscope. The cells were detached from the plate, and their shapes had shrunk and become rounded, leading to an increase in the intercellular spaces. Interestingly, an annexin V/7-AAD assay showed, for the first time, that CC-LAAO stimulated a caspase-independent apoptosis in human glioblastoma U87 cells. However, the other SV-LAAOs induced apoptotic-cell death in tumor cells through the intrinsic and/or extrinsic pathways mediated by a caspase-dependent mechanism [36,37,38,39].

Unfortunately, z-VAD-fmk co-treatment was unable to prevent apoptosis induced in U87 cells. However, the CC-LAAO-apoptosis induction was dependent on oxidative stress because it had been partially prevented by the addition of the NAC. Further investigations are required to evaluate the mechanism underlying the CC-LAAO-induced apoptosis. Indeed, different signaling pathways may lead to this caspase-independent apoptosis.

Through this study, we confirmed that SV-LAAOs are involved in the pathogenesis of snakebite envenomation, as previously reported [28] by inducing necrosis and inflammation in vital organs of the prey. However, low concentrations of CC-LAAO appeared to be safe in the experimental animals. In addition, taken altogether, our results confirmed that CC-LAAO mediates its cytotoxicity via the induction of apoptosis that may presumably be mediated by the release of H_2_O_2_, which agrees with previous reports [12,40,41]. Therefore, additional research into the anti-tumor effects of SV-LAAO via proapoptotic mechanism was indicated by this data. Since LAAO were shown to be safe at low concentrations in our study, it may have therapeutic potential.

## 4. Conclusions

These findings support the importance of the study of these enzymes, not only for a better understanding of their role in ophidian envenomation mechanisms, but also due to their biotechnological activities as potential therapeutic agents.

## 5. Materials and Methods

### 5.1. Biological and Chemical Material

CC-LAAO was purified as described previously [13]. Dulbecco’s Modified Eagle Medium (DMEM), fetal calf serum (FCS) and penicillin/streptomycin were purchased from GIBCO (Cergy-Pontoise, France). Catalase (EC 1.11.1.3, from bovine liver) and N-acetylcysteine (NAC) were purchased from Sigma.

### 5.2. Cells

U87 cells were kindly provided by the Pr. José Luis (Institut de Neurophysiopathologie & Aix-Marseille Université, Faculté de Pharmacie, Marseille, France).

### 5.3. In Vivo Assessment of CC-LAAO Biological Effects on Mice Models

#### 5.3.1. Lethal Dose determination

Lethality of the purified enzyme was tested by intraperitoneal (i.p) injection of different doses of CC-LAAO (10–120 µg), dissolved in 100 µL phosphate-buffered saline (pH = 7.2). Six Swiss albino mice were used for each dose and observed for 48 h for mortality. The control animals received only 100 µL of phosphate-buffered saline.

#### 5.3.2. Assessment of CC-LAAO toxicity

##### In Vivo Experimental Procedure

Swiss albino male mice (6–8 weeks old) were purchased by the animal unit of the Pasteur Institute of Tunis. The mice of 20 ± 2 g were housed for ten days and then randomly divided into six cages of eight mice each under a controlled temperature (22–25 °C) and relative humidity of 40–70%. Animals had ad libitum access to purified water and a standard pellet diet. All the procedures were in accordance with the guidelines for ethical conduct in the care and use of animals.

Groups of mice were named as follows:

- Group 1 (CTR−): received 100 µL of phosphate-buffered saline (PBS) 1×.
- Group 2 (CTR+): Mice received *Cerastes cerastes* crude venom at a dose of 6 µg in 100 µL PBS, which represented half of LD_50_ on Swiss mice [42].
- Group 3 (M1): received CC-LAAO at 1 µg/mL
- Group 4 (M2): received CC-LAAO at 2.5 µg/mL
- Group 5 (M3): received CC-LAAO at 10 µg/mL
- Group 6 (M4): received CC-LAAO at 50 µg/mL.

The concentrations of CC-LAAO used for this study were chosen after the lethal dose determination (data not published).

*Cerastes* (CC) venom, CC-LAAO molecule, and the vehicle were administered via intraperitoneal route at 09:00 a.m. Each mouse underwent one injection with a time interval of 24 h.

All procedures were completed after the approbation of institutional biomedical ethics committee (no. 2015/14/E/FST) and were in accordance with the guidelines for ethical conduct in the care and use of animals.

Mice were observed individually for general behavior, toxic symptoms, and mortality to detect possible health problem, necrosis, or hemorrhage. Blood samples were collected from facial veins of each mouse after 1 h, 6 h, and 24 h, using capillary tubes containing heparin as an anticoagulant, for biochemical parameters study. Mice were euthanized by cervical dislocation, and several organs (i.e., brain, lungs, kidney, and liver) were excised and examined macroscopically. The organs were then fixed in 10% buffered formalin for histopathological examination.

##### Biochemical Parameters Study

Plasma samples were obtained by blood centrifugation at 3000× *g* for 10 min and stored at −20 °C, until use. The activities of hepatic enzymes, such as aspartate aminotransferase (AST), alanine aminotransferase (ALT), and lactate dehydrogenase (LDH) were assessed with commercially available diagnostic kits supplied by Bio Maghreb Laboratories (Tunis, Tunisia). Enzymatic activity was expressed in international units per liter (UI/L). Creatinine level was determined by the method described by Bartels et al. [43].

##### Histological Examination

The organs placed in formalin were washed and then dehydrated by alcohol. Paraffin-embedded sections were cut at 5–6 μm thickness and stained with hematoxylin (H) and eosin (E) for microscopic examination at 400× magnification.

##### Edema-Inducing Activity Assay

Edema-inducing activity was assessed as described by Ali et al. [29] with some modifications. The paws of the Swiss albino mice were injected with the different doses of CC-LAAO (0–24 μg in 50 μL PBS), five mice in each group. As a negative control, an equal volume of normal saline was injected into the contralateral paw of a mouse. After 2 h, the thickness of the swelling paws was measured with a vernier caliper. The minimum edema-forming dose (MED) was defined as the CC-LAAO dose inducing 20% increase in the thickness of the swelling paws.

##### Hemorrhagic Activity Assay

One-day-old fertile eggs obtained from a local hatchery were incubated at 37 °C. The eggs were cracked on day 4, following a standard method [44], and incubated until day 6. Discs of 2 mm diameter cut from filter paper (Whatman) were impregnated with various concentrations of CC-LAAO. The discs were placed on the yolk-sac membrane over a major bilateral vein. Control experiments were performed with the buffered saline solution. After 6 h, hemorrhagic corona formation was observed and photographed with a digital camera at 10× magnification.

### 5.4. Antitumor Activity of CC-LAAO on In Vitro Models

#### 5.4.1. Cell Culture and Cell Growth Conditions

The cell line used was U87 human brain astroblastoma, which is a glioblastoma (GBM). Cells were cultured in DMEM supplemented with 10% FCS. Cells were kept at 37 °C in a humidified atmosphere of 5% CO_2_ in air.

#### 5.4.2. Cell Morphology Analysis and Viability Assay

Cell viability was assessed by the MTT 3-(4,5–DImethylthiazol-2-yl)-2,5-Diphenyl tetrazolium Bromide) assay, as described previously by Mosmann [45]. The cells were treated for 24 h with CC-LAAO at different concentrations (0–5 µg/mL) at 37 °C, and under an atmosphere of 5% CO_2_. The cell morphology was examined and recorded under an inverted-phase-contrast microscope (Leica, Mannheim, Germany). MTT solution (500 µg/mL, final concentration) was added to the wells and incubated for 4 h. Then, 100 µL of DMSO was added in each well, and absorbance was recorded at 560 nm. The percentages of cell viability were used to calculate the IC_50_ value. Mock-treated culture cells were used as a negative control.

To examine the effect of antioxidants on the cytotoxicity induced by CC-LAAO, the viability assay was performed by the co-treatment of U87 cells with CC-LAAO and 100 µg/mL of catalase (EC 1.11.1.6; from bovine liver) or N-acetylcysteine (NAC), for 30 min at room temperature. Catalase is an efficient scavenger of H_2_O_2_ that protects cells against oxidative stress. NAC inhibits reactive-oxygen-species (ROS) production, and it is commonly used to identify and assess ROS inducers.

#### 5.4.3. Apoptosis Quantification by Flow Cytometry Analysis

Phosphatidylserine externalization was determined by flow cytometry using a dead cell apoptosis kit with annexin V FITC and 7-AAD (Molecular Probes, Eugene, OR, USA). U87 tumor cells were treated with different concentrations of CC-LAAO (0.1–2 µg/mL) for 24 h. Mock-stimulated culture cells were used as negative controls, and the positive controls received TNF-related apoptosis-inducing ligand (TRAIL) (0.1 µM for 6 h). Experiments with a pan-caspase inhibitor, z-VAD-FMK (20 µM), or necroptosis inhibitor, Necrostatin (40 µM) or NAC (1 mM), were also performed. Cells were washed twice with PBS, resuspended in a working solution containing 7-AAD (5 μg/mL) and annexin V-FITC (0.25 μg/mL), incubated for 15 min at 4 °C, and analyzed in a FACSCanto flow cytometer (Becton, Dickinson and Company, Franklin Lakes, NJ, USA) using Diva software. Approximately 1 × 10^6^ cells were analyzed for each treatment.

The results were expressed as a percentage of annexin V and/or 7-AAD-stained cells. Data were analyzed using GraphPad Prism software, version 5.0 for Windows.

### 5.5. Statistical Analyses

Data of in vivo study were statistically analyzed using the Student’s *t*-test (one-way ANOVA) by Statistica software for Windows 7.0 to determine significant differences between groups; *p* values less than 0.05 were considered significant. The values were expressed as means ± SD.

For in vitro study, statistical analyses were performed by one-way analysis of variance (ANOVA) combined with Tukey’s post-test to compare all treatments to the negative controls, using the GraphPad Prism Software (version 5 for Windows, GraphPad Software Inc., San Diego, CA, USA). *p* < 0.05 was considered significant.

## Figures and Tables

**Table 1:** Effect of CC-LAAO molecules on creatinine rate in mice after 1 h, 6 h, and 24 h of treatment.

	CTR−	CTR+	CC-LAAO			
			1 µg/mL	2.5 µg/mL	10 µg/mL	50 µg/mL
**1 h**	0.56 ± 0.07	0.815 ± 0.78 *	0.66 ± 0.06	0.63 ± 0.07	0.89 ± 0.09 *	0.965 ± 0.09 **
**6 h**	0.63 ± 0.06	1.05 ± 0.098 **	0.891 ± 0.08 *	0.899 ± 0.06 *	1.002 ± 0.09 **	1.23 ± 0.3 **
**24 h**	0.63 ± 0.07	1.35 ± 0.58 ***	0.963 ± 0.07 *	0.963 ± 0.08 *	1.263 ± 0.1 **	1.55 ± 0.78 ***

CTR−: negative control group; CTR+: mice treated by crude venom (6 µg: the half of LD_50_); M1: mice received CC-LAAO at 1 µg/mL; M2: mice received CC-LAAO at 2.5 µg/mL; M3: mice received CC-LAAO at 10 µg/mL; and M4: mice received CC-LAAO at 50 µg/mL. Data were presented as mean ± SEM, (*n* = 6). * *p* < 0.05, ** *p* < 0.01, and *** *p* < 0.001, versus negative control.

## Data Availability

The data presented in this study are available in this article.
